# Deubiquitinase UCHL1 promotes angiogenesis and blood–spinal cord barrier function recovery after spinal cord injury by stabilizing Sox17

**DOI:** 10.1007/s00018-024-05186-3

**Published:** 2024-03-13

**Authors:** Jiaxing Wang, Chengyue Ji, Wu Ye, Yuluo Rong, Xuhui Ge, Zhuanghui Wang, Pengyu Tang, Zheng Zhou, Yongjun Luo, Weihua Cai

**Affiliations:** 1 Department of Orthopedics, First Affiliated Hospital of Nanjing Medical University, Nanjing, 210029 Jiangsu China; 2 Department of Orthopedics, The Fourth Affiliated Hospital of Soochow University, Suzhou, 215123 Jiangsu China; 3 Department of Emergency Medicine, The First Affiliated Hospital of Nanjing Medical University, Nanjing, 210029 Jiangsu China; 4 Department of Orthopaedics, Centre for Leading Medicine and Advanced Technologies of IHM, Division of Life Sciences and Medicine, The First Affiliated Hospital of USTC, University of Science and Technology of China, Hefei, 230001 Anhui China

**Keywords:** Spinal cord injury, Blood–spinal cord barrier, Sox17, UCHL1, Ubiquitination

## Abstract

Improving the function of the blood–spinal cord barrier (BSCB) benefits the functional recovery of mice following spinal cord injury (SCI). The death of endothelial cells and disruption of the BSCB at the injury site contribute to secondary damage, and the ubiquitin–proteasome system is involved in regulating protein function. However, little is known about the regulation of deubiquitinated enzymes in endothelial cells and their effect on BSCB function after SCI. We observed that Sox17 is predominantly localized in endothelial cells and is significantly upregulated after SCI and in LPS-treated brain microvascular endothelial cells. In vitro Sox17 knockdown attenuated endothelial cell proliferation, migration, and tube formation, while in vivo Sox17 knockdown inhibited endothelial regeneration and barrier recovery, leading to poor functional recovery after SCI. Conversely, in vivo overexpression of Sox17 promoted angiogenesis and functional recovery after injury. Additionally, immunoprecipitation-mass spectrometry revealed the interaction between the deubiquitinase UCHL1 and Sox17, which stabilized Sox17 and influenced angiogenesis and BSCB repair following injury. By generating UCHL1 conditional knockout mice and conducting rescue experiments, we further validated that the deubiquitinase UCHL1 promotes angiogenesis and restoration of BSCB function after injury by stabilizing Sox17. Collectively, our findings present a novel therapeutic target for treating SCI by revealing a potential mechanism for endothelial cell regeneration and BSCB repair after SCI.

**Supplementary Information:**

The online version contains supplementary material available at 10.1007/s00018-024-05186-3.

## Introduction

Spinal cord injury (SCI) is a severe injury to the central nervous system (CNS) caused by trauma, tumors, or inflammation. It disrupts the integrity and continuity of the spinal cord, resulting in motor, sensory, and autonomic dysfunction [1]. Although SCI is not usually fatal, it imposes substantial socioeconomic stress on patients and their families [2]. While progress has been made in understanding the molecular mechanisms of SCI, the clinical translation of this knowledge is unsatisfactory, and effective treatments are still lacking. Currently, the primary focus of treatment is managing the symptoms of SCI. Therefore, further exploration of pathophysiological changes that occur after SCI and identification of effective therapeutic targets are urgently needed for the management of SCI.

Primary SCI refers to the direct disruption of the physiological structures of the spinal cord. In contrast, secondary SCI is indirect damage to the spinal cord tissue caused by edema, ischemia, or inflammation [3]. The blood–spinal cord barrier (BSCB) is a barrier system composed of endothelial cells, pericytes, and glial cells that regulates the molecular exchange between circulating blood and the spinal cord, thereby maintaining CNS homeostasis [4, 5]. After SCI, a large number of endothelial cells in the center of the injury lesion die, leading to rapid disruption of the BSCB. This disruption results in edema, inflammation, neuron death, and glial cell activation [5, 6]. Increasing evidence suggests that BSCB destruction is a major contributing factor to the progression of spinal cord nerve injury. Therefore, targeting BSCB dysfunction after SCI may be a crucial direction for future SCI treatment [7].

The SRY-box (Sox) family of transcription factors is evolutionarily conserved and involved in regulating various biological functions, including development, tissue differentiation, and cell type specification [8]. Sox17 belongs to the SoxF subgroup and possesses a transcriptional activation structural domain sequence in addition to the HMG structural domain [9, 10]. Numerous studies have shown that Sox17, as an endothelial cell-specific transcription factor, plays a pivotal role in endothelial and hematopoietic cell lineage development. It regulates arterial development, induces angiogenesis in tumors, participates in the conversion of fibroblasts to reparative endothelial cells, promotes endothelial cell regeneration in endotoxemia models, and modulates various signaling molecules, such as Notch, TGF-β, Wnt, and vascular endothelial growth factor [11–14]. The stability and activation of Sox17 are primarily influenced by post-translational modifications, including phosphorylation, ubiquitination, SUMOylation, and acetylation [15]. However, the specific regulatory mechanisms of Sox17 after SCI remain unclear and warrant further investigation.

Ubiquitination modifications are among the most common post-translational modifications of proteins, and they play a crucial role in various biological activities by regulating protein stability, signal transduction, and other processes. In the CNS, ubiquitination modifications are widespread [16, 17]. Similar to other post-translational modifications, ubiquitination is a reversible process that can be reversed by certain proteases known as deubiquitinating enzymes (DUBs) [18]. Ubiquitin C-terminal hydrolase L1 (UCHL1), a member of the DUB family, exhibits high specificity and is widely distributed in the brain and spinal cord [19, 20]. Due to its small molecular weight, compact structure, and ability to cross the BSCB, UCHL1 has been considered a potential biomarker for CNS injury [21]. Despite extensive research on UCHL1 in neurodegenerative diseases, its functional role in acute CNS injury, particularly in the regulation of endothelial cells and BSCB after SCI, remains unknown.

The E3 ubiquitin ligase TRIM30 has been previously reported to ubiquitinate Sox17 [15]; however, the effect of the deubiquitinase family on Sox17 remains poorly understood. To address this gap, we generated conditional knockout mice for UCHL1 and established in vivo and in vitro SCI models to investigate whether UCHL1 influences the ubiquitination level of Sox17. Our findings demonstrate that UCHL1 plays an essential regulatory role in angiogenesis and BSCB repair by targeting Sox17 after SCI. This study reveals a novel mechanism of endothelial cell regeneration and BSCB repair following SCI, potentially opening avenues for new therapeutic targets for SCI.

## Methods

### Animals

UCHL1^fl/fl^ (ubiquitin carboxy-terminal hydrolase L1, MGI: 103149, Stock No: CKOCMP-22223-Uchl1-B6J-VA) and Cdh5(PAC)-Cre (cadherin 5, MGI: 7543919, Stock No: C001023) mice on a C57BL/6J background were purchased from Cyagen Biosciences (Cyagen Biosciences, Guangzhou, China). Cdh5-Cre, as the most widely used endothelial cell-specific Cre, has been extensively utilized in endothelial cell research. To delete UCHL1 in endothelial cells, we crossed UCHL1^fl/fl^ mice with Cdh5-Cre mice (Figure S4). In this study, UCHL1^fl/fl^ mice were defined as controls, whereas mice with specific knockout of UCHL1 in endothelial cells were defined as CKO mice. All experiments were approved by the animal committee of First Affiliated Hospital of Nanjing Medical University.

### Mice model of SCI

The mice SCI model was constructed as previously described [22]. Briefly, female mice were anesthetized by intranasal inhalation of 2.0% isoflurane (RWD Life Sciences, China). After skin preparation, laminectomy was performed at the T8 level to fully expose the dorsal aspect of the spinal cord, and an SCI model was established by dropping a 5 g rod from a height of 6.5 cm using a spinal cord percussion device (RWD Life Sciences, China). The success of SCI was verified by observing body tremors, tail wagging, and contraction of the hind limbs and body of the mice. Postoperative assisted urination was performed daily until bladder function returned to normal.

### Immunofluorescence staining assays

After anaesthetising the mice, the thoracic cavity was opened to expose the heart, and the mice were perfused with cold 0.9% saline from the left heart, followed immediately by 4% paraformaldehyde. Spinal cord sections were removed and post-fixed overnight in 4% paraformaldehyde at 4 ℃. They were then dehydrated in a sucrose gradient solution of 20% and 30% and cut into 10 μm thick cryosections. For immunofluorescence staining, tissue sections were fixed with 4% paraformaldehyde for 15 min, permeabilized with 0.3% Triton X-100 for 15 min, blocked with 5% bovine serum albumin (BSA) for 1 h, and incubated with primary antibodies overnight at 4 ℃. After thorough washing with PBS, cell samples or tissue sections were co-incubated with secondary antibodies (1:200, Jackson ImmunoResearch, USA) for 2 h at room temperature. Sections were counterstained with DAPI and photographed using a Thunder Imager (THUNDER DMi8, LEICA, Germany). Fluorescence quantification was performed using ImageJ software.

### Antibodies

The primary antibodies used for Immunofluorescence staining in this study were anti-CD31 (1:300, Abcam, ab222783), anti-ZO-1 (1:300, Abcam, ab221547), anti-Occludin (1:300, Abcam, ab216327), anti-NeuN (1:300, Abcam, ab104224), anti-GFAP (1:1000, Abcam, ab7260), anti-Sox17 (1:100, Santa Cruz Biotechnology, sc-130295), and anti-CD68 (1:300, Abcam, ab283654). The secondary antibodies used for immunofluorescence staining in this study were Alexa Fluor® 488 AffiniPure™Goat Anti-Rabbit IgG (H + L)(1:300, Jackson, 111-545-003) and Alexa Fluor® 594 AffiniPure™Goat Anti-Mouse IgG (H + L)(1:300, Jackson, 115-585-003). Primary antibodies used for western blot studies were anti-ZO-1 (1:1000, Abcam, ab221547), anti-Occludin (1:1000, Abcam, ab216327), anti-β-actin (1:2000, Servicebio, Wuhan, China), anti-Sox17 (1:500, Santa Cruz Biotechnology, sc-130295; 1:1000, Proteintech, Wuhan, China, 24903-1-AP), anti-CD31(1:1000, Abcam, ab222783), anti-UCHL1 (1:1000, Abcam, ab108986), anti-Flag-Tag (1:2000, Cell Signaling Technology, #14793), anti-Myc-Tag (1:2000, Cell Signaling Technology, #9402), and anti-HA-Tag (1:2000, Cell Signaling Technology, #3724). The secondary antibodies used for western blot studies were HRP conjugated Goat Anti-Rabbit IgG (H + L) (1:5000, Servicebio, GB23303) and HRP conjugated Goat Anti-Mouse IgG (H + L) (1:5000, Servicebio, GB23301).

### Functional behavior analysis

The mice were housed in the Animal Experiment Center of Nanjing Medical University, with ad libitum access to food and water, and maintained in a 12 h/12 h light/dark cycle. Functional behavior tests were performed at 1, 3, 7, 14, 21, and 28 d post-injury. Before the tests, the mice were allowed to acclimate to the experimental environment and instruments, and their motor functions were assessed by three trained experimentalists in a double-blind manner.

### Basso Mouse Scale (BMS) score

The severity of motor dysfunction in mice after SCI was rated using the BMS score on a scale of 0–9, with 9 indicating near normal activity and 0 indicating no ankle motion.

### Rotarod test

The overall motor ability and coordination of mice were assessed 28 d post-injury using a rotarod. The mice were placed on a speed-adjustable (0–40 rpm) rotarod, and the speed of the rod and the duration of the mice were recorded until they fell off. The mice underwent one practice session followed by two test sessions, and the results were averaged.

### Footprint analysis

Following a previously described method [23], mice had different colored dyes applied to their front and hind paws. They were then placed on a runway with white paper on the bottom and encouraged to walk forward. Footprint pictures were collected, and step and stride lengths were measured to assess motor function.

### Electromyography analysis

Motor evoked potentials (MEPs) were recorded using electromyography 28 d post-injury. After the mice were anesthetized, a stimulating electrode was placed at the rostral end of the exposed spinal cord, recording electrode was inserted deep into the flexor muscle of the biceps femoris, reference electrode was placed in the distal tendon, and grounding electrode was placed subcutaneously. Stimulation-evoked potentials at 0.5 mA, 0.5 ms, and 1 Hz were used, and amplitudes and latencies were collected to evaluate motor function recovery.

### Assessment of BSCB permeability

Mice in the sham-operated group and mice with SCI were intravenously injected with Evans blue dye (200 μl). Three hours later, the mice were euthanized, and spinal cord tissues were obtained. After the pictures of spinal cord tissues were retained, 10 μm thick tissue sections were prepared. Evans blue leakage was recorded using fluorescence microscopy (THUNDER DMi8, LEICA, Germany).

### Quantitative reverse transcription PCR

Cellular and tissue RNA were extracted using TRIzol reagent (Invitrogen), and the RNA was reverse transcribed to cDNA using the PrimeScript RT kit (TaKaRa, Japan). Quantitative reverse PCR was performed using the MiScript SYBR Green PCR kit (QIAGEN, Germany). The relative expression of RNA was calculated using the 2^-ΔΔCT^ method after normalizing to the internal reference. The primer sequences used in this study were as follows: Sox17: forward 5′-GATGCGGGATACGCCAGTG-3′ and reverse 5′-CCACCTCGCCTTTCACCTTTA-3′; GAPDH: forward 5′- GGAGAGTGTTTCCTCGTCCC-3′ and reverse 5′-ATGAAGGGGTCGTTGATGGC-3′.

### Cell culture

HEK293T cells and bEnd.3 brain microvascular endothelial cells were obtained from the Cell Bank of the Chinese Academy of Sciences (Shanghai, China). The cells were cultured in DMEM containing 10% FBS and 1% penicillin–streptomycin at 37 ℃ and 5% CO_2_. The cells were placed in either 6-well plates at a density of 3 × 10^5^ or 24-well plates at a density of 1.5 × 10^5^. To maintain their growth, the media was refreshed every 2 days. For the subsequent experiments, the cells were allowed to grow to the desired density. It should be noted that only cell lines within 5–10 generations were used for this study.

### Western blot analysis

Total protein was extracted from cells or tissues using a total protein extraction kit, and protein concentration was measured using the BCA method (Thermo Fisher Scientific) following the manufacturer’s instructions. Proteins were separated by SDS-PAGE and transferred to PVDF membranes. After being blocked in 5% BSA for 1 h at room temperature, PVDF membranes were incubated overnight with the primary antibody on a 4 ℃ shaker. On the second day, after washing with TBST, the PVDF membranes were incubated with horseradish peroxidase-coupled anti-rabbit or anti-mouse IgG antibodies (1:2000, Thermo Fisher, USA) for 1 h. Finally, immunolabeled bands were visualized using enhanced chemiluminescence reagents (Thermo Fisher), and protein expression was quantified using ImageJ software.

### Immunoprecipitation

Following a previously described method [23], whole protein lysates were incubated with control IgG and 20 μl protein A/G plus-agarose (Santa Cruz Biotechnology, USA) at 4 ℃ for 30 min. The lysates were then centrifuged to remove nonspecific bound proteins. Subsequently, the lysates were co-incubated with the specific primary antibody for 1 h at 4 ℃, followed by co-incubation with 20 μl protein A/G plus agarose overnight at 4 ℃. The immune complexes were collected by centrifugation, washed five times, and subjected to immunoblot analysis.

For silver staining and immunoprecipitation-mass spectrometry (IP-MS), Flag-Sox17 was expressed in vascular endothelial cells, and whole protein lysates were extracted. The lysates were then co-incubated with BeyoMag™ anti-Flag magnetic beads (Beyotime, China) and eluted using 3 × Flag peptide (Beyotime) to remove IgG light and heavy chains. The eluate was separated using SDS-PAGE for mass spectrometry (MS; Thermo Fisher Scientific, USA) and silver staining (Beyotime) according to the manufacturer’s instructions.

### Transepithelial electrical resistance (TEER) measurement

As previously described [6], bEnd.3 cells were seeded in a culture chamber with 0.4 μm pore size (Millipore, USA). Measurements were performed after the cells had reached 100% confluence. Before each measurement, the cells were pre-equilibrated with HBSS for 30 min. Resistance values were measured using a Millicell® ERS instrument (Millipore) following the manufacturer’s instructions.

### Permeability assay

Endothelial cells were seeded in the aforementioned culture chambers. After reaching 100% confluence, 100 µl of FITC-dextran (4 kDa, Sigma Aldrich, USA)-containing medium was added to the upper chamber, whereas 500 µl of the normal medium was added to the lower chamber. After 2 h of incubation in the dark, the medium from the lower chamber was collected for fluorescence measurement using a Microplate Reader.

### ShRNA and plasmid construction

Control and Sox17-targeted short hairpin RNAs (NC-KD and Sox17-KD, respectively) were constructed by Genebay Biotech (Nanjing, China). Control plasmids, full-length Sox17 expression plasmid (FL 1-414), Flag-Sox17 structural domain 1-135 and 136-414 plasmids, HA-Ub expression plasmids (HA-WT-Ub, HA-K63-Ub, and HA-K48-Ub), and full-length UCHL1 expression plasmids (WT or C90S mutants) were also constructed by Genebay Biotech. Transfection of brain microvascular endothelial cells and HEK 293T cells was performed using Lipofectamine 3000 reagent according to the manufacturer’s instructions.

### Adeno-associated virus

To increase the exogenous expression of Sox17 and UCHL1 in vivo, pAAV-PHP.eB-Tie2-Sox17-3 × FLAG-WPRE (AAV-Sox17) (Obio Technology, Shanghai, China), pAAV-PHP.eB-Tie2-UCHL1-3 × FLAG-WPRE (AAV-UCHL1), and pAAV-PHP.eB-Tie2-3 × FLAG-WPRE as a control vector (AAV-Con) were used. The AAV-PHP.eB vector (AAV-shSox17) or control (AAV-shCon) was further constructed to deplete local Sox17 in vivo by expressing short hairpin RNA. After SCI, the viral vector (5 × 10^9^ vg; 1 µl) was injected into the spinal cord at the site of injury using a 10 µl Hamilton Neuros syringe connected to a micro syringe pump controller (RWD) at a rate of 200 nl/min.

### Statistical analysis

The results are presented as mean ± standard deviation, and all statistical analyses were performed using GraphPad Prism (version 8.0, GraphPad Software Inc., USA). The means of two groups were compared using independent-samples Student’s t-test, while comparisons among more than two groups were conducted using one-way or two-way analysis of variance followed by post hoc Bonferroni correction. Statistical significance was set at P < 0.05 (two-tailed) for all tests.

## Results

### Disruption of the BSCB after SCI

**Fig. 1:**
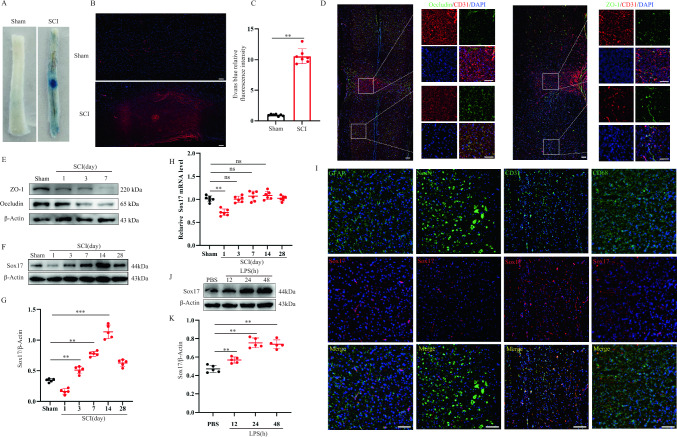
Disruption of the BSCB after SCI. **A** Representative images of spinal cord after Evans Blue dye injection at day 7 post-injury. **B** Immunofluorescence analysis of spinal cord after Evans Blue dye injection at day 7 post-injury (Scale bar = 200 μm, Objective: 10X, n = 7). **C** Quantification of Evans Blue dye fluorescence intensity. We conducted a semi-quantitative analysis of the fluorescence intensity of Evans blue dye by measuring the average gray value, where the gray value of each pixel in a single-channel image represents the fluorescence intensity at that specific point. The average gray value was calculated by dividing the integrated density by the area. **D** Representative co-immunostaining of CD31 and TJs markers in the injured spinal cord at day 7 post-injury, with the adjacent area and lesion enlarged (Scale bar = 200 μm, Objective: 10X, n = 6). **E** Expression levels of spinal TJs proteins were examined by western blot at indicated time after SCI (n = 5). **F** Representative immunoblots showing levels of Sox17 protein in injured spinal cord at Days 1, 3, 7, 14, and 28 post-injury (n = 5). **G** Quantification of relative levels of Sox17 protein. **H** Expression of Sox17 mRNA in injured spinal cord examined by qPCR (n = 6). **I** Immunofluorescence analysis of the injured spinal cord revealed that Sox17 is predominantly expressed by endothelial cells (CD31), with minor expression observed in astrocytes (GFAP). However, no expression of Sox17 was detected in macrophages/microglia (CD11b) or neurons (NeuN). (Scale bar = 100 μm, Objective: 20X). **J** Representative immunoblots showing levels of Sox17 protein in endothelial cells after LPS treatment at different time points (n = 7). **K** Quantification of relative levels of Sox17 protein. *p < 0.05; **p < 0.01. The data is analyzed using Student’s t-test

To examine the barrier function of the BSCB after SCI, we administered Evans blue dye to mice in the sham and SCI groups. Significant dye leakage was observed in the injury center of mice in the SCI group but not in the sham group (Fig. 1A). Immunofluorescence results showed that the dye was localized within the blood vessels in the Sham group, whereas it leaked significantly in the epicenter of the SCI mice (Fig. 1B, C). The expression of TJ proteins that maintain the barrier function of the BSCB was further examined after injury. The immunofluorescence results showed that ZO-1 and Occludin co-localized significantly less with CD31 in the center of the injury area compared with that in the non-injured area (Fig. 1D). Additionally, the immunoblotting results demonstrated a significant decrease in the protein levels of the TJs after SCI (Fig. 1E; Figure S1). These findings suggest that the integrity of the BSCB is disrupted after SCI.

Endothelial cells are a major component of the BSCB, and their modification is particularly crucial as they are extensively lost following SCI, resulting in abnormal barrier function. Sox17 has been extensively studied in recent years as an endothelial-specific transcription factor in vascular regeneration and maintenance of arterial identity.

[14, 24]. However, the function of Sox17 after SCI has not been elucidated. Therefore, we focused on exploring the potential regulatory mechanisms of Sox17 after SCI. We found that Sox17 expression was significantly decreased at 1 d post-SCI and then consistently increased, reaching a maximum at 14 d post-injury (Fig. 1F, 1G). Notably, *Sox17* mRNA levels were not significantly altered after SCI (Fig. 1H). Given the presence of multiple cell types in spinal cord tissue, including astrocytes (GFAP^+^), macrophages/microglia (CD11b^+^), neurons (NeuN^+^), and endothelial cells (CD31^+^), we further investigated whether Sox17 expression was predominantly localized in vascular endothelial cells. Immunofluorescence results showed that Sox17 was predominantly expressed by CD31^+^ vascular endothelial cells, with minor expression observed in astrocytes (Fig. 1I). Brain vascular endothelial cells were treated with LPS to mimic the inflammatory microenvironment of SCI in vivo, and Sox17 protein expression levels in endothelial cells were examined. The results showed that the Sox17 protein was significantly upregulated in response to injury (Fig. 1J, 1K). These results suggest that the BSCB is disrupted after SCI and that elevated Sox17 expression may play a key role in endothelial cells.

### In vivo knockdown or overexpression of endothelial Sox17 influences functional recovery in mice

**Fig. 2:**
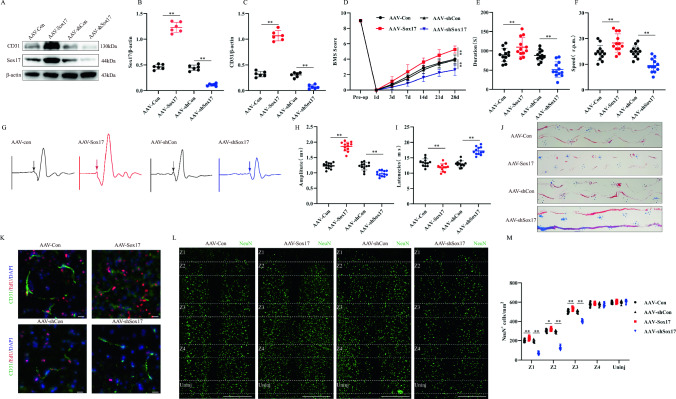
In vivo knockdown or overexpression of endothelial Sox17 influence functional recovery in mice.** A** Representative immunoblots showing levels of CD31 and Sox17 protein in injured spinal cord at Day 14 post-injury in AAV-Con, AAV-Sox17, AAV-shCon and AAV-shSox17 group (n = 6). **B**, **C** Quantification of relative levels of Sox17 and CD31 protein. (D) BMS Scoring in different groups up to Day 28 post-injury (n = 8). **E**, **F** Rotarod tests in different groups at Day 28 post-injury (n = 12). **G** MEP analysis was used as electrophysiological assessment in different groups at Day 28 post-injury (n = 12, arrows indicate onset of evoked potential). **H**, **I** Quantification of peak-to-peak MEP amplitudes and latencies in different groups. **J** Representative footprints of animal walking 28 days post SCI. Compared to the control group, mice in the AAV-Sox17 group exhibited longer hindlimb stride length and reduced step width, indicating better motor function. Conversely, mice in the AAV-shSox17 group showed increased step width, suggesting impaired motor function. (n = 6, Blue: front paw print; red: hind paw print). **K** Representative immunofluorescence images of endothelial cells proliferation by double-staining EdU and CD31 in different groups at Day 14 post-injury (Scale bar = 50 μm, Objective: 40X, n = 6). **L** Representative immunofluorescence images of NeuN^+^ neurons in Z1–Z4 zones adjacent to central lesion core in different groups at Day 14 post-injury (Scale bar = 1000 μm, Objective: 10X, n = 8). **M** Quantification of NeuN^+^ neurons in Z1–Z4 zones. *p < 0.05; **p < 0.01. The data is analyzed using Student’s t-test

To investigate the potential role of Sox17 in endothelial cells after SCI, we injected adeno-associated viruses that specifically overexpressed or knocked down Sox17 (AAV-Sox17 or AAV-shSox17, respectively) in vascular endothelial cells immediately after SCI. Immunoblotting results showed that AAV-Sox17 significantly increased the protein expression of Sox17 and CD31, whereas AAV-shSox17 inhibited their expression (Fig. 2A–C). BMS scoring, rotarod test, electromyography analysis, and footprint analysis showed that compared with that in controls, overexpression of Sox17 promoted functional recovery after injury in mice, whereas knockdown of Sox17 in endothelial cells inhibited functional recovery after injury in mice (Fig. 2D–J). More EdU^+^/CD31 positive cells were observed with the administration of AAV-Sox17 compared to controls. In contrast, AAV-ShSox17 reduced EdU^+^/CD31 positive cells, suggesting that Sox17 stimulated endothelial cell proliferation (Fig. 2K). Sox17 is hypothesized to promote vascular regeneration in vivo, thereby protecting the surrounding tissues from further damage. To test our speculation, we performed staining of neuronal markers to further assess the survival of neurons in specific regions (Z1–Z4), as previously described [25]. Overexpression of Sox17 significantly increased the number of neurons in the Z1–Z2 region, whereas knockdown of Sox17 reduced the number of neurons in this region. The Z3–Z4 and undamaged regions showed no difference in the mice (Fig. 2L, M). These results suggest that endothelial cell-specific expression of Sox17 promotes endothelial cell regeneration and functional recovery after injury in mice, whereas knockdown of Sox17 is detrimental to functional recovery.

### In vitro knockdown or overexpression of Sox17 influences endothelial cell regeneration and barrier function

**Fig. 3:**
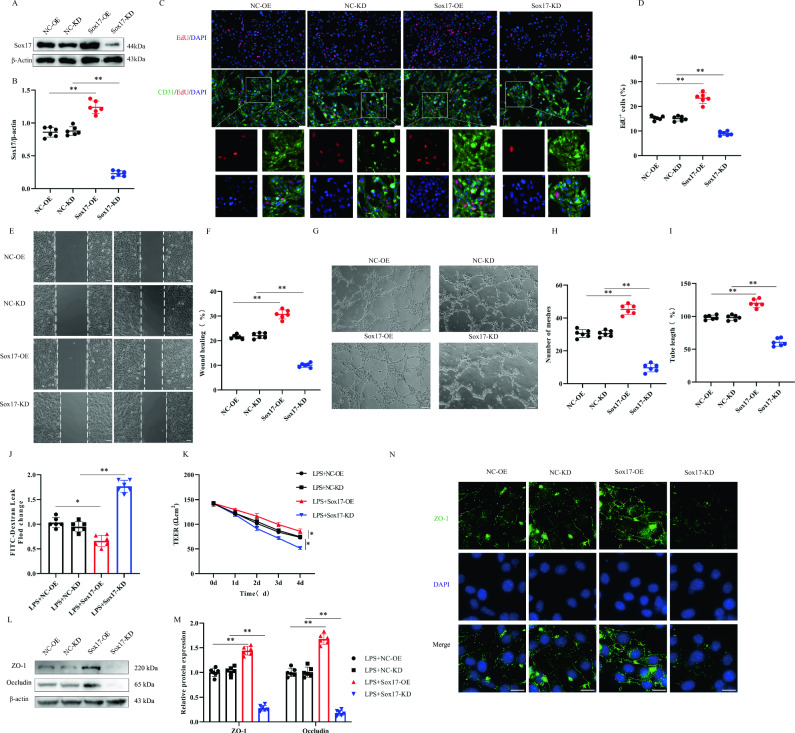
In vitro knockdown or overexpression of Sox17 influence endothelial cell regeneration and barrier function. **A** Representative immunoblots showing levels of Sox17 protein in endothelial cell in NC-OE, Sox17-OE, NC-KD and Sox17-KD group (n = 6). **B** Quantification of relative levels of Sox17 protein. **C** Representative immunofluorescence images of endothelial cells proliferation by double-staining EdU and CD31 in different groups (Scale bar = 100 μm, Objective: 20X, n = 6). **D** Quantification of EdU^+^ cells. **E** Representative images of the migratory capacity of endothelial cells in different groups (Scale bar = 100 μm, Objective: 10X, n = 6). **F** Quantification of wound healing. **G** Representative images of the tube-forming capacity of endothelial cells in different groups (Scale bar = 100 μm, Objective: 10X, n = 6). **H**, **I** Quantification of mesh numbers and tube length. **J** FITC-Dextran leak and **K** TEER value was used to evaluate the effect of barrier function of endothelial cells (n = 6). **L** Representative immunoblots showing endothelial TJs protein levels in different groups treated with LPS (n = 6). **M** Quantification of relative levels of TJs protein. **N** Immunofluorescence detection of ZO-1 in endothelial cells (Scale bar = 10 μm, Objective: 40X, n = 6). *p < 0.05; **p < 0.01. The data is analyzed using Student’s t-test

To further investigate the potential function of Sox17 in vascular endothelial cells, we used adenoviral vectors to knock down or overexpress Sox17 (Sox17-KD or Sox17-OE, respectively) in vitro and verified the knockdown and overexpression effects using immunoblotting (Fig. 3A, B). Through EdU, scratch, and tube formation assays, we observed that overexpression of Sox17 promoted endothelial cell proliferation, migration, and angiogenesis, whereas its knockdown inhibited endothelial cell proliferation, migration, and tube formation (Fig. 3C–I). To assess the effect of Sox17 on endothelial cell barrier function in an inflammatory microenvironment mimicking post-SCI conditions, we stimulated endothelial cells with LPS. The results demonstrated that overexpression of Sox17 significantly reduced FITC leakage, decreased endothelial resistance loss, and protected endothelial barrier function, whereas its knockdown further deteriorated endothelial barrier function (Fig. 3J, K).

We examined the expression of TJs, which play a major role in endothelial barrier function, and both immunoblotting and immunofluorescence results showed that Sox17 reduced the loss of TJ proteins after injury and facilitated the recovery of barrier function (Fig. 3L–N). Collectively, these findings suggest that overexpression of Sox17 promotes endothelial cell regeneration and barrier function recovery, whereas its knockdown inhibits endothelial cell regeneration and impairs barrier function.

### Sox17 interacts with UCHL1

**Fig. 4:**
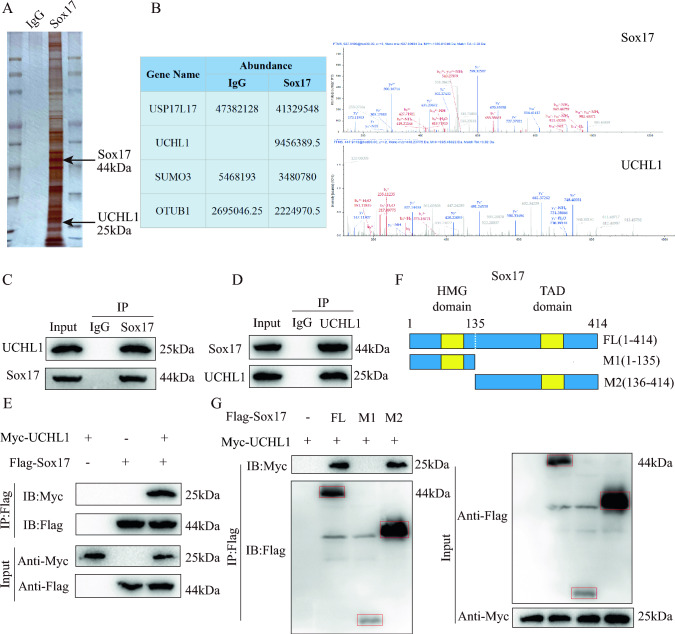
Sox17 interacts with UCHL1. **A** Silver-stained gel of endothelial cells proteins coimmunoprecipitated using anti-Flag magnetic beads. **B** IP/MS analysis to determine which proteins bind to Sox17 showed UCHL1 to be a specific binding protein. **C** Endogenous protein interactions in endothelial cells lysates assessed by immunoprecipitation with anti-Sox17 or anti-IgG and evaluated by immunoblotting with anti-UCHL1 (n = 3). **D** Endogenous protein interactions in endothelial cells lysates assessed by immunoprecipitation with anti-UCHL1 or anti-IgG and evaluated by immunoblotting with anti-Sox17 (n = 3). **E** Demonstration of exogenous protein interactions in HEK 293 T cells. Lysates from HEK 293 T cells transfected with Flag-tagged Sox17 and Myc-tagged UCHL1 plasmids were immunoprecipitated with anti-Flag, followed by immunoblotting with anti-Myc and anti-Flag (n = 3). **F** Schematic representations of Myc-tagged full-length (FL) UCHL1, Flag-tagged full-length (FL) Sox17 and their various deletion mutants. **G** HEK 293 T cells were co-transfected with Myc-UHCL1 and Flag-tagged FL Sox17 or its deletion mutants, and cell lysates were evaluated by immunoprecipitation followed by immunoblotting with anti-Myc and anti-Flag (n = 3)

To explore the potential mechanisms regulating Sox17 expression, we performed immunoprecipitation to extract all Sox17 binding proteins, which were then identified using MS. Considering that the protein expression of Sox17 was altered after SCI without significant changes in mRNA levels (Fig. 1F–H), we hypothesized that post-translational modifications are involved in the altered protein expression of Sox17. In the field of CNS research, ubiquitination is a well-studied post-translational modification. Therefore, we examined the MS results to identify any deubiquitinase expressions associated with Sox17 and found that UCHL1 bound specifically to Sox17 (Fig. 4B). The results of silver staining and MS indicated that UCHL1, a classical deubiquitinating enzyme, was a Sox17-interacting protein (Fig. 4A, B; Figure S3). The IP-MS findings regarding the binding of UCHL1 to Sox17 were validated through co-immunoprecipitation (Co-IP) experiments (Fig. 4C, D). Additionally, we constructed plasmids containing Flag-Sox17 and Myc-UCHL1 and verified their interactions in HEK-293T cells. The co-precipitation of Flag-Sox17 and Myc-UCHL1 was successfully observed as anticipated (Fig. 4E). To identify the specific binding region of UCHL1 to Sox17, we generated both full-length and truncated mutations of flag-tagged Sox17 (Fig. 4F). These constructs were transfected into HEK-293T, followed by Co-IP experiments. Our findings revealed that the amino acid fragment 136-414 of Sox17 could bind to UCHL1 (Fig. 4G). Taken together, these results suggest an interaction between UCHL1 and Sox17.

### UCHL1 modulates Sox17 ubiquitination

**Fig. 5:**
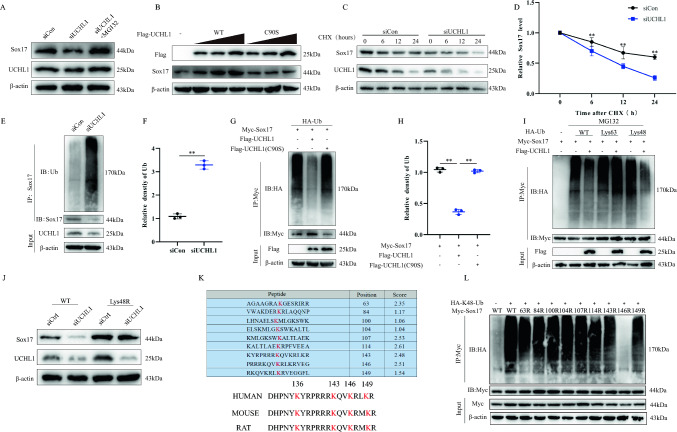
UCHL1 modulates Sox17 ubiquitination. **A** Analysis of UHCL1 and Sox17 in endothelial cells transfected with siUHCL1, with and without treatment with proteasome inhibitor MG132 (n = 3). **B** Increasing amounts of Flag-tagged UHCL1 (WT or C90S mutant) were transfected into HEK 293 T cells, and cell lysates were analyzed by immunoblotting with anti-Sox17 and anti-Flag (n = 3). **C** Sox17 protein levels in siCtrl and siUHCL1 endothelial cells were measured by immunoblotting against Sox17 and UCHL1 in the absence and presence of cycloheximide (10 μg/ml) for the indicated times (n = 3). **D** Quantification of relative levels of Sox17 in the absence and presence of cycloheximide for the indicated times. **E** Lysates from endothelial cells transfected with siCtrl or siUCHL1, followed by treatment with MG132 before harvest, were immunoprecipitated and examined with indicated antibodies (n = 3). **F** Quantification of relative ubiquitin-Sox17 levels. **G** Lysates from HEK 293 T cells transfected with HA-tagged Ub and Myc-tagged Sox17 together with Flag-tagged UHCL1 (WT) or Flag-tagged UHCL1 (C90S) were immunoprecipitated with anti-Myc, followed by immunoblotting with anti-HA and anti-Myc (n = 3). **H** Quantification of relative Ub-Sox17 levels. **I** HEK 293T cells were cotransfected with Myc-Sox17, Flag-UHCL1, and the indicated HA-Ub, Lys48-only, or Lys63-only plasmids, and then the Sox17 ubiquitylation linkage was analyzed (n = 3). **J** HEK293T cells transfected with Ub WT or Ub Lys48R were cultured for 72 h in the presence of siCtrl or siUHCL1. Cell lysates were analyzed by immunoblotting (n = 3). **K** Using bioinformatics we predicted all evolutionarily conserved potential ubiquitinated lysine sites for Sox17. **L** HEK 293T cells were co-transfected with Myc-Sox17 (wild-type or various KR mutants), HA-Ub and then Myc-Sox17 ubiquitylation was analyzed (n = 3). *p < 0.05; **p < 0.01. The data is analyzed using Student’s t-test

The role of UCHL1 as a deubiquitinating enzyme has been demonstrated in several studies [19, 26]. Since UCHL1 interacts with Sox17, we investigated the role of UCHL1 in the regulation of Sox17 in endothelial cells. The knockdown of UCHL1 was observed to significantly reduce Sox17 protein levels compared with that in controls, but the addition of the proteasome inhibitor MG132 reversed the reduction of Sox17 protein (Figs. 5A, S2A). We further explored the effect of UCHL1 on Sox17 protein stability by expressing UCHL1 or its C90S mutant (reduced catalytic activity) in a dose gradient (Figs. 5B; S2B, 2C). We found that overexpression of UCHL1, but not its mutant, increased Sox17 protein expression. To investigate the effect of UCHL1 on Sox17 protein half-life, we added the protein synthesis inhibitor CHX and observed that UCHL1 knockdown significantly reduced the protein half-life of Sox17 compared with that in the control group (Fig. 5C, D). As ubiquitination modification plays a crucial role in protein regulation, we investigated whether UCHL1 regulates the degradation of Sox17 by modulating its ubiquitin levels. Knockdown of UCHL1 in endothelial cells significantly increased the level of Sox17 ubiquitination and decreased its protein expression compared with those in controls (Fig. 5E, F). The regulation of Sox17 ubiquitination by UCHL1 was further validated in HEK-293T cells (Fig. 5G, H). Through transfection of Myc-Sox17, HA-Ub, Flag-UCHL1, and Flag-UCHL1 (C90S) plasmids, followed by immunoprecipitation, we observed that UCHL1 expression reduced the ubiquitination level of Sox17. However, this effect was abolished by UCHL1 (C90S). K48 ubiquitination is associated with the proteasomal degradation pathway, while K63 ubiquitination is associated with signaling transduction. Additionally, K6, K11, K27, K29, and K33 are associated with various other biological activities. We found that UCHL1 specifically cleaves the Lys48-linked polyubiquitin chain of Sox17 but has no significant effect on the Lys63-linked polyubiquitin chain of Sox17 (Fig. 5I). To validate these results, we overexpressed Lys48-resistant ubiquitin (Lys48R) in HEK-293T cells and observed that Lys48R ubiquitin expression reversed the UCHL1 knockdown-induced reduction in Sox17 protein expression (Fig. 5J). Finally, we investigated potential ubiquitylation sites on the Sox17 protein. Employing the protein/peptide ubiquitination prediction tool available on 'GPS-Uber (biocuckoo.cn)', we input the mouse Sox17 FASTA format sequence to identify potential ubiquitination sites for Sox17 (Fig. 5K). Our predictions encompassed all evolutionarily conserved potential ubiquitination sites for Sox17, including K63, K84, K100, K104, K107, K114, K143, K146, and K149. By constructing a series of lysine mutant plasmids and co-transfecting with HA-Ub in HEK-293T cells, we observed a significantly lower level of ubiquitylation after mutation of lysine 146 of Sox17 (Fig. 5L). Taken together, UCHL1 regulates Sox17 stability by deubiquitinating it.

### Conditional knockdown of UCHL1 in endothelial cells prevents endothelial cell regeneration and functional recovery after injury

**Fig. 6:**
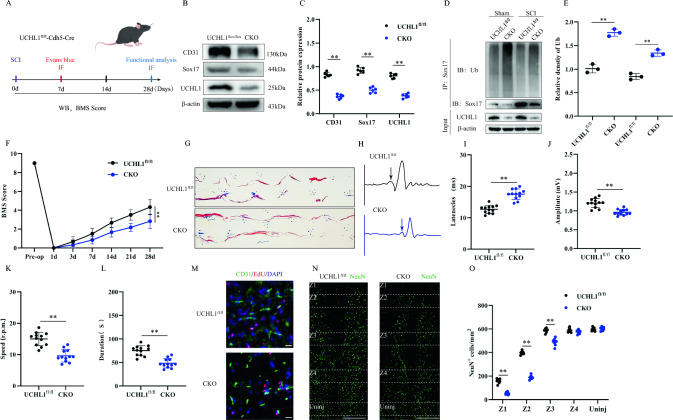
Conditional knockdown of UCHL1 in endothelial cells prevents endothelial cell regeneration and functional recovery after injury. **A** Schematic diagram of the experiment time. **B** Representative immunoblots showing levels of CD31, UHCL1 and Sox17 protein in injured spinal cord at Day 14 post-injury in UCHL1^fl/fl^ and CKO group (n = 6). **C** Quantification of relative levels of Sox17, UHCL1 and CD31 protein. **D** Spinal cord lysates from UCHL1^fl/fl^ and CKO mice after SCI were immunoprecipitated with antiSox17, Ub-Sox17 was examined by immunoblotting (n = 3). **E** Quantification of relative Ub-Sox17 levels. **F** BMS Scoring in UCHL1^fl/fl^ and CKO group up to Day 28 post-injury (n = 6). **G** Representative footprints of animal walking 28 days post SCI. Compared to UCHL1^fl/fl^ mice, CKO mice exhibited longer hindlimb step width, decreased stride length, and noticeable dragging, indicating poorer motor function.(n = 6, Blue: front paw print; red: hind paw print). **H** MEP analysis was used as electrophysiological assessment in UCHL1^fl/fl^ and CKO group at Day 28 post-injury (n = 12). **I**–**J** Quantification of peak-to-peak MEP amplitudes and latencies. **K**, **L** Rotarod tests in UCHL1^fl/fl^ and CKO group at Day 28 post-injury (n = 12). **M** Representative immunofluorescence images of endothelial cells proliferation by double-staining EdU and CD31 in UCHL1^fl/fl^ and CKO group at Day 14 post-injury (Scale bar = 50 μm, Objective: 40X, n = 6). **N** Representative immunofluorescence images of NeuN^+^ neurons in Z1–Z4 zones adjacent to central lesion core in UCHL1^fl/fl^ and CKO group at Day 14 post-injury (Scale bar = 1000 μm, Objective: 10X, n = 6). **O** Quantification of NeuN^+^ neurons in Z1–Z4 zones. *p < 0.05; **p < 0.01. The data is analyzed using Student’s t-test

We generated endothelial cell-specific UCHL1 knockout (UCHL1 CKO) mice to evaluate the function of UCHL1 in endothelial cells after SCI. We performed a series of experimental analyses on UCHL1^fl/fl^ (control) and UCHL1 CKO mice at different time points after SCI (Fig. 6A). Immunoblotting results showed that UCHL1 protein expression was reduced in CKO mice compared with that in UCHL1^fl/fl^ mice, along with the reduced expression of Sox17 and CD31 proteins observed after injury (Fig. 6B, C), indicating that UCHL1 knockdown may inhibit angiogenesis in vivo. We further examined the regulation of ubiquitination of Sox17 by endogenous UCHL1 following SCI. Compared with UCHL1^fl/fl^ mice, CKO mice exhibited significantly increased levels of Sox17 ubiquitination and decreased Sox17 protein expression in both the sham and SCI groups (Fig. 6D, E), suggesting that UCHL1 may stabilize Sox17 through deubiquitination modifications. Motor functions after SCI, quantified using BMS scoring, revealed that CKO mice had worse functional recovery than UCHL1^fl/fl^ mice (Fig. 6F). The footprint analysis (Fig. 6G) and rotarod test (Fig. 6K, L) validated these results, demonstrating that UCHL1 CKO mice showed worse gait recovery and inadequate motor coordination. We conducted electrophysiological recordings of MEPs in UCHL1^fl/fl^ and CKO mice, which showed that MEPs in CKO mice after SCI had lower amplitude and longer latencies (Fig. 6H–J). Endothelial cell death and disruption of barrier function are common after SCI. Endothelial regeneration and barrier reconstruction are essential for functional recovery. Based on the involvement of UCHL1 in Sox17 ubiquitination modification, we hypothesized that UCHL1 also plays a regulatory role in endothelial cell regeneration after SCI. Immunofluorescence results demonstrated that conditional knockdown of UCHL1 significantly reduced the number of EdU^+^ endothelial cells after injury (Fig. 6M). Furthermore, staining of surviving neurons in specific regions (Z1–Z4) of the spinal cords of UCHL1 CKO and UCHL1^fl/fl^ mice with the neuron-specific marker NeuN revealed a significantly reduced number of viable NeuN^+^ neurons in Z1–Z3 in CKO mice compared with that in UCHL1^fl/fl^ mice (Fig. 6N–O). Collectively, these results suggest that conditional deletion of UHCL1 impairs endothelial cell regeneration and motor recovery after SCI.

### UCHL1 promotes endothelial cell regeneration and functional recovery in mice by increasing Sox17 levels after SCI

**Fig. 7:**
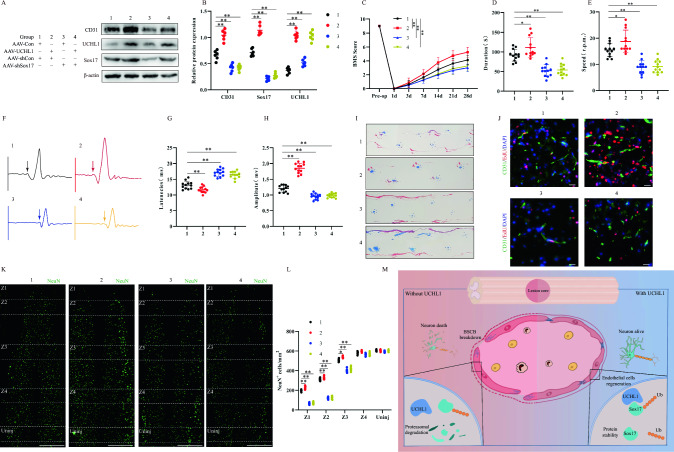
UCHL1 promotes endothelial cell regeneration and functional recovery in mice by increasing Sox17 levels after SCI. **A** Detailed information of test groups in this part of experiment. Representative immunoblots showing levels of CD31, UHCL1 and Sox17 protein in injured spinal cord at Day 14 post-injury in different groups (n = 6). **B** Quantification of relative levels of Sox17, UHCL1 and CD31 protein. **C** BMS Scoring in different groups up to Day 28 post-injury (n = 8). **D**, **E** Rotarod tests in different groups at Day 28 post-injury (n = 12). **F** MEP analysis was used as electrophysiological assessment in different groups at Day 28 post-injury (n = 12). **G**, **H** Quantification of peak-to-peak MEP amplitudes and latencies. **I** Representative footprints of animal walking 28 days post SCI. Compared to the control group, mice in the AAV-UCHL1 group exhibited longer hindlimb stride length and significantly reduced step width, indicating better motor function. However, co-administration of AAV-shSox17 abolished the beneficial effects of AAV-UCHL1. (n = 6, Blue: front paw print; red: hind paw print). **J** Representative immunofluorescence images of endothelial cells proliferation by double-staining EdU and CD31 in different groups at Day 14 post-injury (Scale bar = 50 μm, Objective: 40X, n = 6). **K** Representative immunofluorescence images of NeuN^+^ neurons in Z1–Z4 zones adjacent to central lesion core in different groups at Day 14 post-injury (Scale bar = 1000 μm, Objective: 10X, n = 6). **L** Quantification of NeuN^+^ neurons in Z1–Z4 zones. **M** Mechanistic diagram of UCHL1 regulation of angiogenesis and barrier function through deubiquitination of Sox17. UCHL1 promotes vascular neovascularization, barrier function restoration and motor function recovery by stabilizing Sox17 and upregulating its expression after spinal cord injury. *p < 0.05; **p < 0.01. The data is analyzed using Student’s t-test

Having established that conditional knockdown of UCHL1 inhibits functional recovery in mice, we investigated the role of UCHL1 in the selective targeting of Sox17 through reversion experiments. To assess the associated protein expression, we performed immunoblotting after injecting AAV-UCHL1, AAV-shSox17, and their respective controls to overexpress UCHL1 or knockdown Sox17 in mice (Fig. 7A, B). The recovery of motor function in mice after AAV delivery was quantified using BMS scoring, and we showed that AAV-shSox17 abolished the recovery of motor function in AAV-UCHL1 mice (Fig. 7C). The rotarod test (Fig. 7D, E) and footprint analysis (Fig. 7I) further validated that Sox17 knockdown reversed the recovery of gait and motor coordination in UCHL1-overexpressing mice. Additionally, UCHL1 overexpression reduced the latency and increased the amplitude of MEPs in mice, whereas further knockdown of Sox17 reversed this effect (Fig. 7F–H). We evaluated endothelial cell proliferation and the number of surviving neurons through immunofluorescence (Fig. 7J–L). The results demonstrated that AAV-UCHL1 administration promoted endothelial cell regeneration and increased the number of surviving neurons in mice, whereas the knockdown of Sox17 reversed this effect. These findings suggest that UCHL1 promotes angiogenesis and enhances motor function recovery in mice by increasing Sox17 expression (Fig. 7M).

## Discussion

In this study, we propose an innovative role for Sox17 expression in the regeneration of vascular endothelial cells and the repair of the BSCB after SCI; this regulatory role has not been reported to date. Increased Sox17 protein expression following SCI promotes endothelial cell regeneration and barrier repair. The deubiquitinase UCHL1 reduces the ubiquitination of Sox17 in vivo, thereby promoting its stability after SCI. Conditional knockdown of UCHL1 in endothelial cells reduces the expression of Sox17 protein after injury and inhibits functional recovery. Therefore, Sox17 may serve as a potential target for the treatment of SCI.

Vascular disruption and dysfunction after SCI are considered key factors in the failure of spinal cord neural tissue repair and secondary tissue damage [27]. Degenerative changes in endothelial cells and blood vessels can occur as early as 30 min after SCI and continue to worsen over the first 3 d, particularly at the site of injury [28]. Shear forces mechanically disrupt blood vessels, leading to increased permeability (BSCB dysfunction) and subsequent secondary injuries [29]. Fassbender et al. [27] demonstrated that enhanced functional angiogenesis or vascular protection improves motor recovery. Ni et al. [30] showed that deletion of UTX/KDM6A promotes SCI recovery by regulating vascular regeneration, highlighting the clinical promise of angiogenesis-targeted therapies in SCI. Therefore, a comprehensive study of the potential mechanisms underlying vascular endothelial cells and BSCB regeneration and repair after injury is crucial for SCI treatment.

Sox17 is a key regulator of oligodendrocyte lineage differentiation in the CNS [31–33]. However, emerging evidence has classified Sox17 as an endothelial cell-specific transcription factor, with multiple regulatory roles in the endothelial cell lineage [12, 34]. The function of Sox17 in endothelial cells after SCI remains unclear and requires further exploration. We first examined the protein expression levels of Sox17 at different time points after SCI and observed the lowest expression on day 1, followed by a consistent increase, with the highest expression on day 14. The exact mechanism of Sox17 downregulation in the early phase of SCI is unclear, and the time point of its downregulation suggests that the acute phase response to SCI may play a potential role. This downregulation may be the result of activation of specific signalling pathways triggered by cellular stress, inflammation or injury, and further studies are still needed. Interestingly, no significant changes in *Sox17* mRNA levels were observed after SCI. The discrepancy between Sox17 protein levels and mRNA levels suggests the possibility of post-transcriptional modifications. Additionally, immunofluorescence assays revealed that Sox17 is predominantly expressed in endothelial cells, further supporting its regulatory role in endothelial cell alterations after SCI. Corada et al. [35] demonstrated that Sox17 interacts with the Wnt pathway to regulate blood–brain barrier permeability, and endothelial-specific knockdown of Sox17 leads to increased microcirculatory permeability. Furthermore, activation of Sox17 in an endotoxemia model promotes endothelial cell regeneration and restores vascular homeostasis [14]. Therefore, we investigated the role of Sox17 in endothelial cell regeneration, vascular remodeling, and barrier function after SCI. Through in vitro and in vivo knockdown and overexpression of Sox17, we found that Sox17 overexpression promoted endothelial cell regeneration, barrier repair, and motor function recovery after injury in mice, whereas Sox17 knockdown has the opposite effect.

Although previous studies have focused on the downstream functional effects of Sox17, only a few have explored the underlying reasons for its altered expression. To elucidate the potential mechanism of altered Sox17 expression, we performed an IP-MS assay and identified UCHL1 as an interacting protein of Sox17. We further validated the binding between Sox17 and UCHL1 through endogenous and exogenous Co-IP experiments with deleted fragment structural domain analyses. UCHL1 has been implicated in sperm development [36], breast cancer [37], cardiac hypertrophy [19], and Parkinson’s disease [38], among other disorders. In the CNS, UCHL1 has been identified as a biomarker for acute and chronic neurological injuries [39]. Notably, Sox17 protein levels were elevated after SCI without significant changes in mRNA levels, and UCHL1 serves as a deubiquitinating enzyme. These points imply a potential regulatory role of ubiquitination modifications in Sox17 after SCI. The regulatory role of deubiquitination modifications after SCI has been extensively studied. Rong et al. [23] demonstrated that increased expression of USP11 in neurons after SCI promotes functional recovery after injury by stabilizing Beclin1. The USP1/UAF1 complex improves motor function in mice by stabilizing METTL3 after SCI [40]. Liu et al. [41] provided evidence that the ubiquitination of the transcription factor Sox9 regulates its protein stability. As Sox17 is also a transcription factor, ubiquitination may play a role in the altered expression of Sox17 following SCI. Indeed, Ubiquitination of Sox17 has been reported in thyroid cancer [15]. In our study, we show that the increased protein expression of Sox17 after SCI is mediated by the deubiquitinase UCHL1.

Another notable finding is that conditional knockout of UCHL1 in mice endothelial cells reduces the protein expression of Sox17, inhibits endothelial cell regeneration, and impairs motor function recovery in mice. Conversely, overexpression of Sox17 promotes functional recovery. Considering the highly complex pathological processes in SCI, where numerous signaling molecules play intricate roles, we cannot exclude other regulatory mechanisms of endothelial cells and BSCB function, such as the direct regulatory effect of UCHL1 on endothelial cells. Pan et al. [42] suggested the involvement of UCHL1 in the regulation of human choroidal and retinal endothelial proliferation. Furthermore, UCHL1 reportedly regulates pulmonary vascular endothelial cell permeability in vitro and in vivo [43]. In our study, we focused on considering UCHL1 as a deubiquitinase that acts on Sox17 for its functional role. However, acknowledging that UCHL1 may directly act on endothelial cells after injury to promote endothelial cell regeneration and protect the BSCB function is essential. The regeneration of vascular endothelium and restoration of barrier function after injury involve complex cascades of reactions, and a single isolated mechanism is insufficient to explain this intricate process. Therefore, further research is warranted to explore other potential mechanisms.

## Conclusion

Endothelial cell regeneration, BSCB integrity, and functional recovery after SCI are closely linked. The interaction between UCHL1 and Sox17 plays a crucial role in promoting endothelial cell regeneration and BSCB repair after injury. Our findings provide new therapeutic targets for the treatment of SCI by highlighting the importance of UCHL1-mediated post-translational modifications of Sox17 in endothelial cells.

### Supplementary Information

Below is the link to the electronic supplementary material.

Supplementary file1 (DOCX 1397 KB)

## Data Availability

The data that support the findings of this study are available on request from the corresponding author.

## Abbreviations

BSCB: Blood–spinal cord barrier
SCI: Spinal cord injury
CNS: Central nervous system
TJ: Tight junctions
Sox: SRY-box
UCHL1: Ubiquitin C-terminal hydrolase L1
DUB: Deubiquitinating enzyme
BMS: Basso Mouse Scale
MS: Mass spectrometry
Co-IP: Co-immunoprecipitation
MEP: Motor evoked potential
IP-MS: Immunoprecipitation-mass spectrometry
