# Supplementary material for: Deubiquitinase UCHL1 promotes angiogenesis and blood–spinal cord barrier function recovery after spinal cord injury by stabilizing Sox17
Source: Cell Mol Life Sci. 2024 Mar 13;81(1):137. doi: 10.1007/s00018-024-05186-3 (PMC10937794; doi:10.1007/s00018-024-05186-3)
Supplement: Supplementary file 1 — Supplementary file1 (DOCX 1397 KB) [file 18_2024_5186_MOESM1_ESM.docx]

**Supplementary Materials**


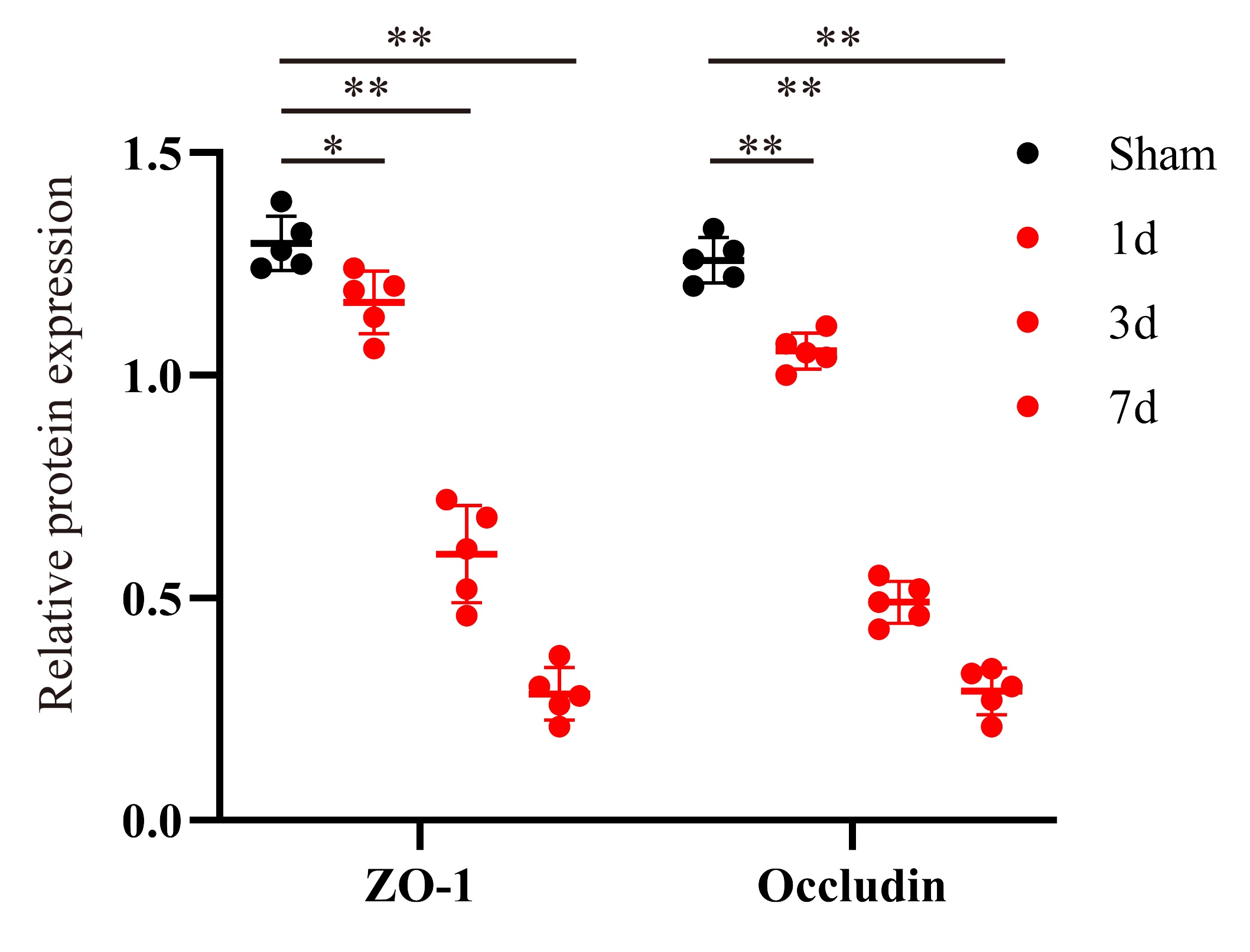


**Figure S1.** Quantification of relative levels of ZO-1 and Occludin protein in Fig 1e.**p* <0.05; ***p* <0.01. The data is analyzed using Student’s t-test.

**
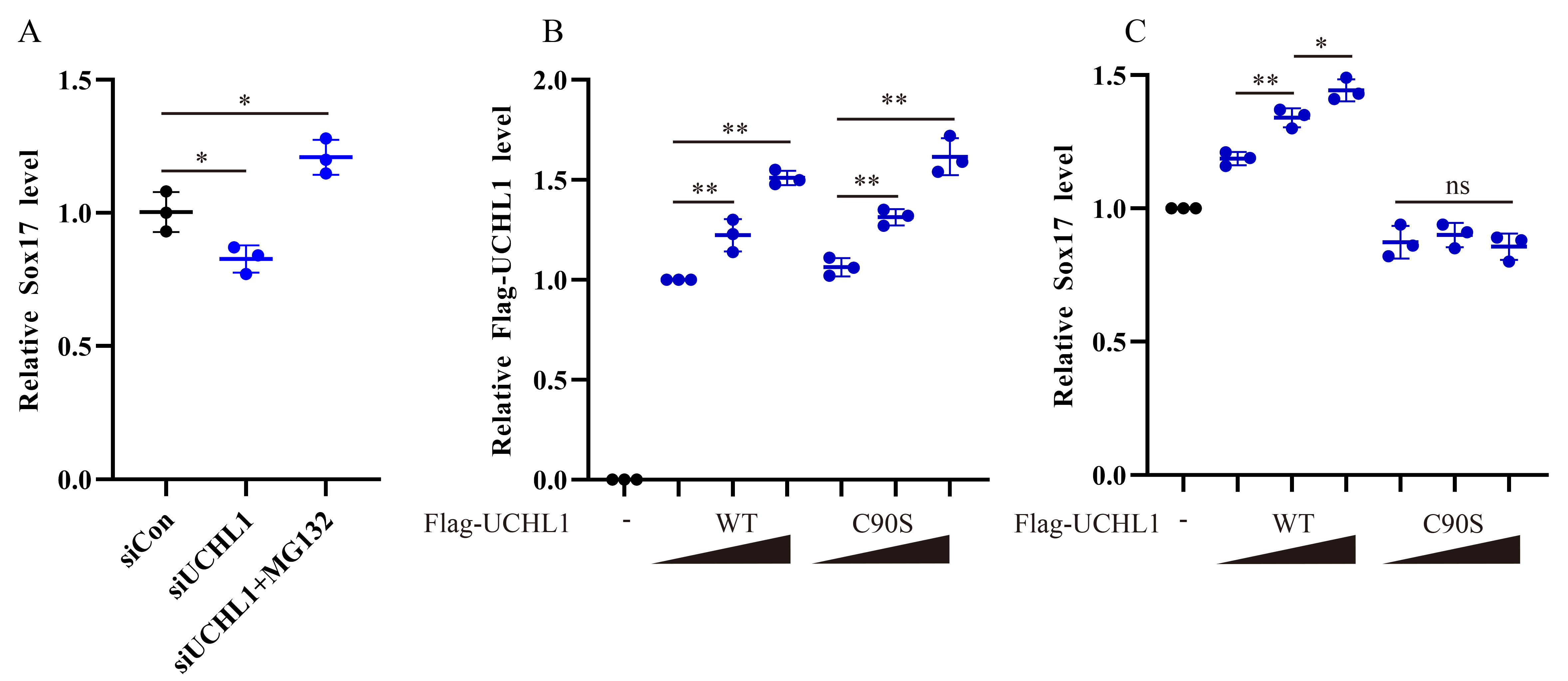
**

**Figure S2.** (A) Quantification of relative levels of Sox17 protein in Fig 5a. (B-C) Quantification of relative levels of Flag-UCHL1 and Sox17 protein in Fig 5b.**p* <0.05; ***p* <0.01. The data is analyzed using Student’s t-test.

UCHL1


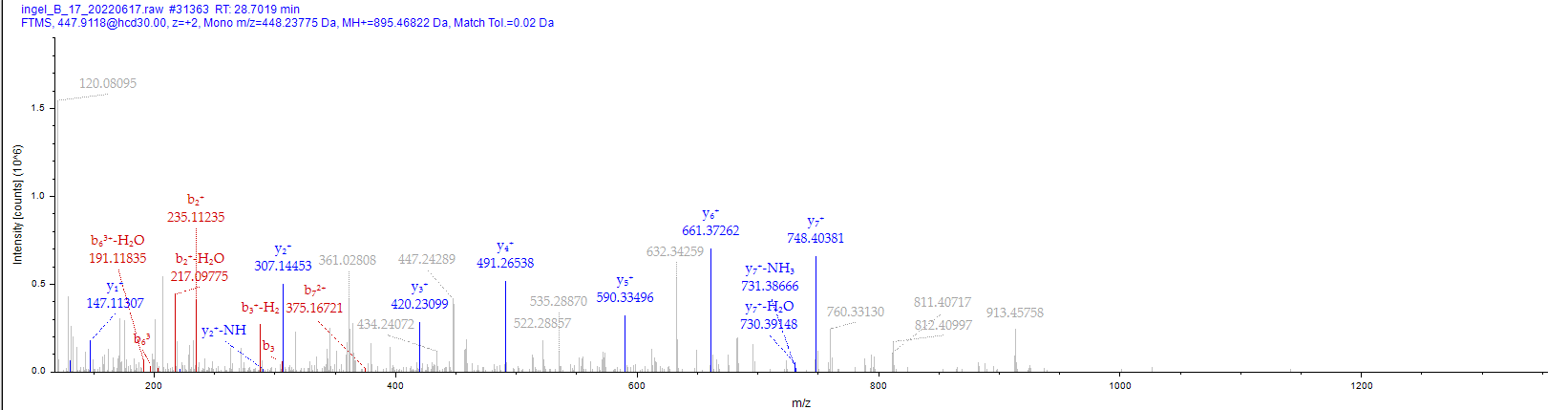


Sox17


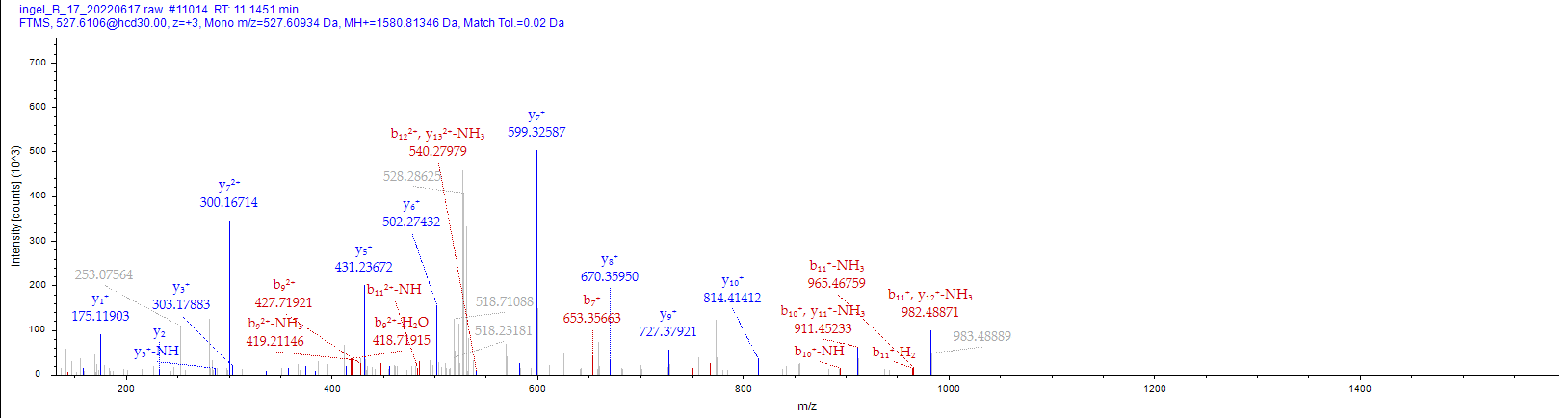


**Figure S3.** IP/MS analysis to determine which proteins bind to Sox17 showed UCHL1 to be a specific binding protein.

**
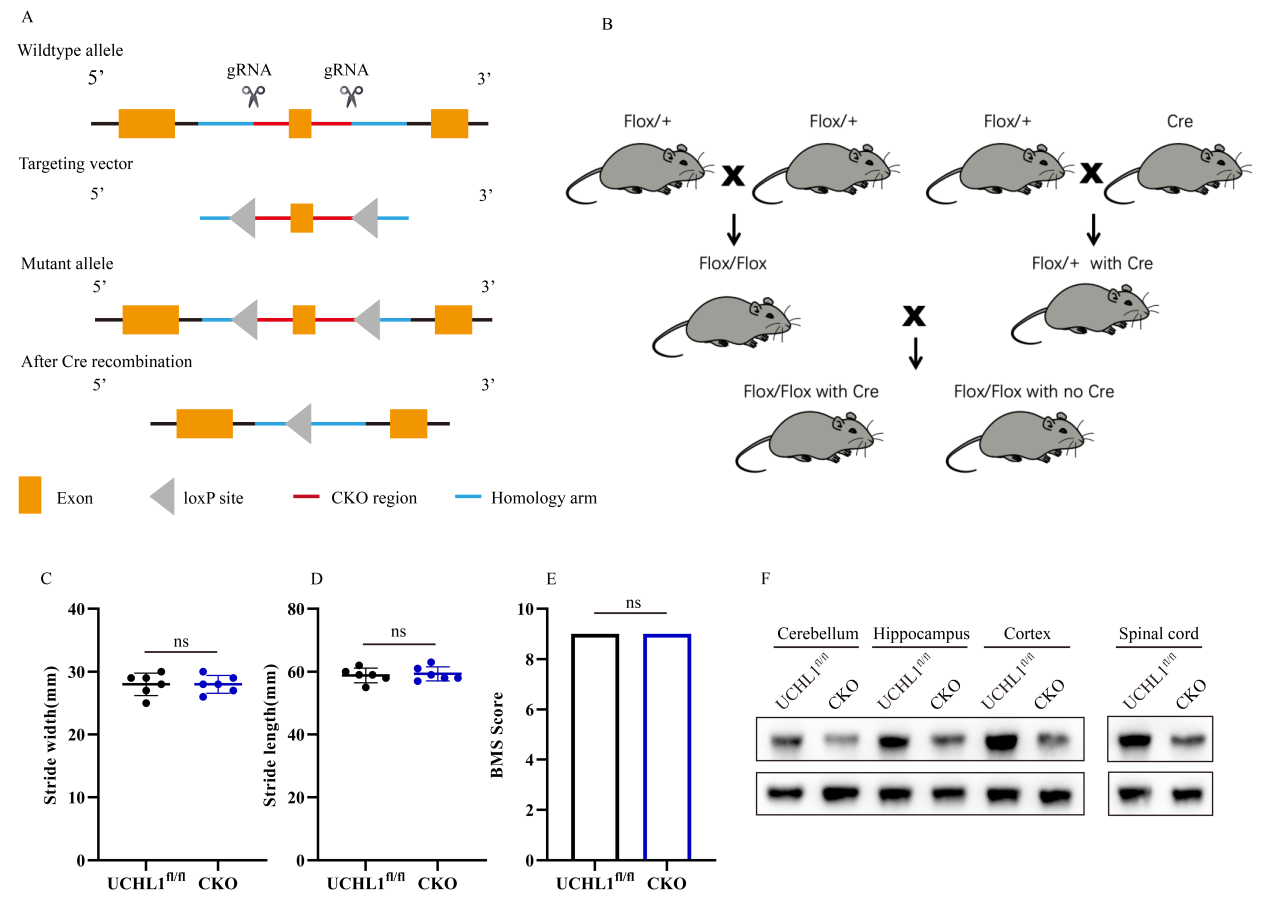
**

**Figure S4.** (A-B) Illustration of transgenes used in this experiment. UCHL1fl/fl-Cdh5(PAC)-Cre mice were generated by crossing mice with UCHL1 floxed with mice with the Cdh5(PAC)-Cre transgene. (C-D) Stride width and stride length of UCHL1fl/fl and CKO mice without SCI (n = 6). (E) BMS score of UCHL1fl/fl and CKO mice without SCI (n = 6). (F) Expression of UCHL1 in the cerebellum, hippocampus, cortex, and spinal cord of UCHL1fl/fl and CKO mice (n = 3). *p <0.05; **p <0.01. The data is analyzed using Student’s t-test.
